# Astroglial Activation by an Enriched Environment after Transplantation of Mesenchymal Stem Cells Enhances Angiogenesis after Hypoxic-Ischemic Brain Injury

**DOI:** 10.3390/ijms17091550

**Published:** 2016-09-14

**Authors:** Sung-Rae Cho, Hwal Suh, Ji Hea Yu, Hyongbum (Henry) Kim, Jung Hwa Seo, Cheong Hoon Seo

**Affiliations:** 1 Department and Research Institute of Rehabilitation Medicine, Yonsei University College of Medicine, Seoul 03722, Korea; srcho918@yuhs.ac (S.-R.C.); onlyjin112@naver.com (J.H.Y.); 2 Brain Korea 21 PLUS Project for Medical Science, Yonsei University College of Medicine, Seoul 03722, Korea; hkim1@yuhs.ac; 3 Rehabilitation Institute of Neuromuscular Disease, Yonsei University College of Medicine, Seoul 03722, Korea; 4 Yonsei Stem Cell Research Center, Avison Biomedical Research Center, Seoul 03722, Korea; 5 Graduate Program of Nano Science and Technology, Yonsei University, Seoul 03722, Korea; hwal@yuhs.ac; 6 Department of Pharmacology, Yonsei University College of Medicine, Seoul 03722, Korea; 7 Department of Rehabilitation Medicine, Hallym University Burn Institute, Burn Center, Hangang Sacred Heart Hospital, Seoul 03722, Korea

**Keywords:** hypoxic-ischemic brain injury, mesenchymal stem cells, enriched environment, angiogenesis, astrocytes

## Abstract

Transplantation of mesenchymal stem cells (MSCs) has paracrine effects; however, the effects are known to be largely limited. Here we investigated the combination effects of cell transplantation and enriched environment (EE) in a model of hypoxic-ischemic brain injury. Brain damage was induced in seven-day-old mice by unilateral carotid artery ligation and exposure to hypoxia (8% O_2_ for 90 min). At six weeks of age, the mice were randomly assigned to four groups: phosphate-buffered saline (PBS)-control (CON), PBS-EE, MSC-CON, and MSC-EE. Rotarod and grip strength tests were performed to evaluate neurobehavioral functions. Histologic evaluations were also performed to confirm the extent of astrocyte activation and endogenous angiogenesis. An array-based multiplex ELISA and Western blot were used to identify growth factors in vivo and in vitro. Two weeks after treatment, levels of astrocyte density and angiogenic factors were increased in MSC-EE mice, but glial scarring was not increased. Eight weeks after treatment, angiogenesis was increased, and behavioral outcomes were synergistically improved in the MSC-EE group. Astrocytes co-cultured with MSCs expressed higher levels of angiogenic factors than astrocytes cultured alone. The mechanisms of this synergistic effect included enhanced repair processes, such as increased endogenous angiogenesis and upregulation of angiogenic factors released from activated astrocytes.

## 1. Introduction

An enriched environment (EE) has been a classic paradigm for studying the effects of a complex combination of physical, cognitive, and social stimulation in rodents [1,2]. An EE including running wheels, novel objects, and social interactions is a model of rehabilitation in rodents. In many studies, an EE has exhibited therapeutic effects such as enhancement of neurogenesis [3], angiogenesis [4], neural plasticity [5], and astroglial activation without glial scarring [3] via secretion of beneficial factors in a normal or injured brain. These effects have been hypothesized to lead to functional recovery [3,4].

Multipotent mesenchymal stem cells (MSCs) derived from adipose are abundantly harvested from lipoaspirates and are not associated with ethical or immunological problems [6,7,8]. Although MSCs can differentiate into cells of the neural lineage in vitro and express neuronal or glial markers in the ischemic brain of animal models [9,10,11,12,13], we previously demonstrated the low survival rate of MSCs and number of differentiated cells into βIII-tubulin^+^ neuronal cells after MSC transplantation into the brain. Indeed, recent evidence suggests that transplanted MSCs also secrete various paracrine factors, including cytokines, chemokines, and growth factors [6,14]. Moreover, MSCs treated group showed transient improvements in neuronal behavioral assessments [3]. These findings suggest that the beneficial effects of MSCs are due to indirect paracrine mechanisms rather than cell replacement and direct tissue regeneration [3,15,16]; thus, transplantation of adipose-derived MSCs may result in limited and transient effects instead of long-lasting permanent improvement [3].

Astrocytes are the most abundant cells in the brain and play a crucial role in the formation of the blood brain barrier (BBB) via their endfeet processes. However, excessive reactivation of astrocytes leads to the retraction of astrocyte endfeet from vessels and increased glial scarring [17,18,19,20]. Park et al. showed that MSCs stabilize the BBB by regulating astrocytic endfeet and vascular endothelial growth factor (VEGF-A) signaling [17]. Other studies have shown that astrocytes secrete glial cell-derived neurotrophic factor (GDNF) [21,22], VEGF [22,23,24], fibroblast growth factor-2 (FGF-2) [22,25], and brain-derived neurotrophic factor (BDNF) [22,26] after MSC transplantation. Therefore, reactive astrocytes promote recovery from injury after MSCs grafting.

We have previously shown that an EE enhances the engraftment of transplanted MSCs by providing a beneficial microenvironment, and that the combination of an EE and MSCs induces astrocyte activation and FGF-2 secretion [3]. In the present study, we further investigated whether astrocytes activated by an EE after MSC transplantation can induce angiogenesis and other processes that cause functional recovery.

## 2. Results

### 2.1. The Combination of an Enriched Environment (EE) and Mesenchymal Stem Cells (MSCs) Induces Astrocyte Activation

**Figure 1:**
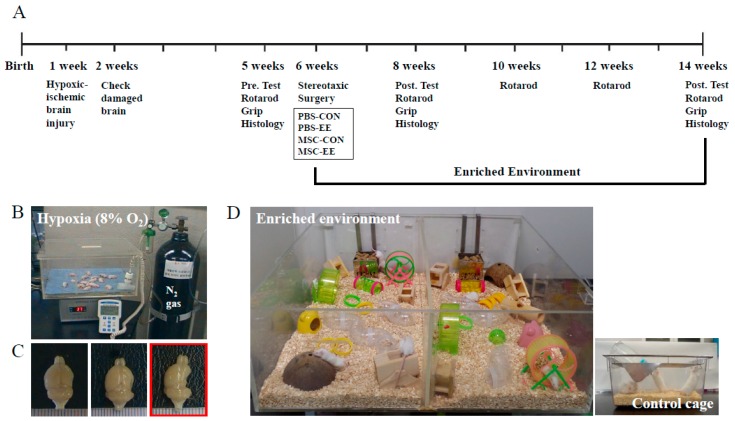
Experimental designs: (**A**) Schematic timeline of the experimental procedures; (**B**) mice were monitored in hypoxic conditions (8% O_2_) controlled with N_2_ gas for 90 min after unilateral carotid artery ligation; (**C**) in the left and middle panels, the damaged brain showed an ipsilateral lesion in the posterolateral hemisphere, while the right panel in the red box shows that severely injured mice were excluded from this study; and (**D**) an enriched environment, including tunnels, shelters, toys, running wheels for voluntary exercise, and social interaction and standard cage at the right bottom.

**Figure 2:**
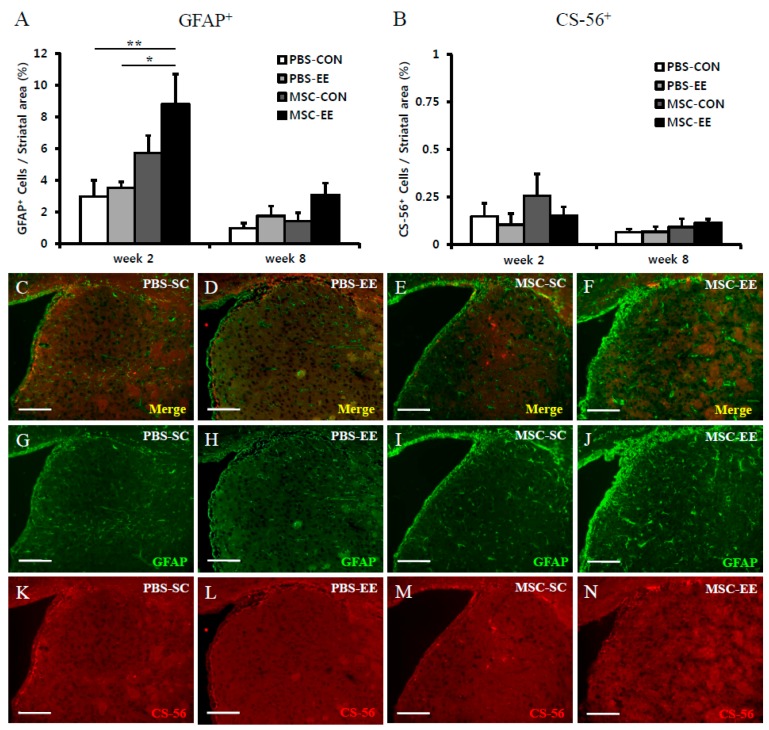
The combination of MSCs and an EE induces astrocyte activation. (**A**–**N**) Two and eight weeks after an EE and MSC transplantation, the amounts of GFAP^+^ cells (green color) and CS-56^+^ cells (red color) were evaluated using the MetaMorph Imaging System. (**A**) The density of striatal GFAP^+^ cells (%) was significantly higher in the MSC-EE mice than in both the PBS-CON and PBS-EE groups two weeks after treatment (* *p* < 0.05 and ** *p* < 0.01, respectively; *n* = 5 each); (**B**) The CS-56^+^ cell density did not differ between groups; (**C**–**N**) Two weeks after an EE and MSC transplantation, representative images are shown. Scale bars = 100 μm. Values represent mean + SEM.

Astrocytes play crucial roles in the recovery of the injured brain. Thus, we evaluated the levels of activated astrocytes after treatment with MSCs and/or an EE in a model of hypoxic-ischemic brain injury (Figure 1). The density of striatal GFAP^+^ cells (%) was calculated as the GFAP^+^ area (in mm^2^) divided by the striatal area (in mm^2^). The GFAP^+^ cell density was significantly increased in the MSC-EE group at two weeks post-treatment (Figure 2A,C–J; *p* < 0.01 and *p* < 0.05 compared with PBS-CON and PBS-EE, respectively). Specifically, the GFAP^+^ cell densities were MSC-EE (8.8% ± 1.8%), MSC-CON (5.7% ± 1.1%), PBS-EE (3.5% ± 0.4%), and PBS-CON (3.0% ± 1.0%). At eight weeks post-treatment, the MSC-EE group exhibited a significant increase (Figure 2A; *p* < 0.05 compared with PBS-CON): MSC-EE (3.1% ± 0.7%), MSC-CON (1.4% ± 0.5%), PBS-EE (1.7% ± 0.6%), and PBS-CON (1.0% ± 0.3%). In addition, the level of the glial scarring marker CS-56 did not differ among the groups (Figure 2B,K–N), demonstrating that the combination of an EE and MSCs does not result in glial scarring, which inhibits neuronal regeneration after injury. This result suggests that an EE was able to sustain endogenous astrocyte activation after transplantation of MSCs in a synergistic manner.

### 2.2. The Combination of an EE and MSCs Enhances Endogenous Angiogenesis

**Figure 3:**
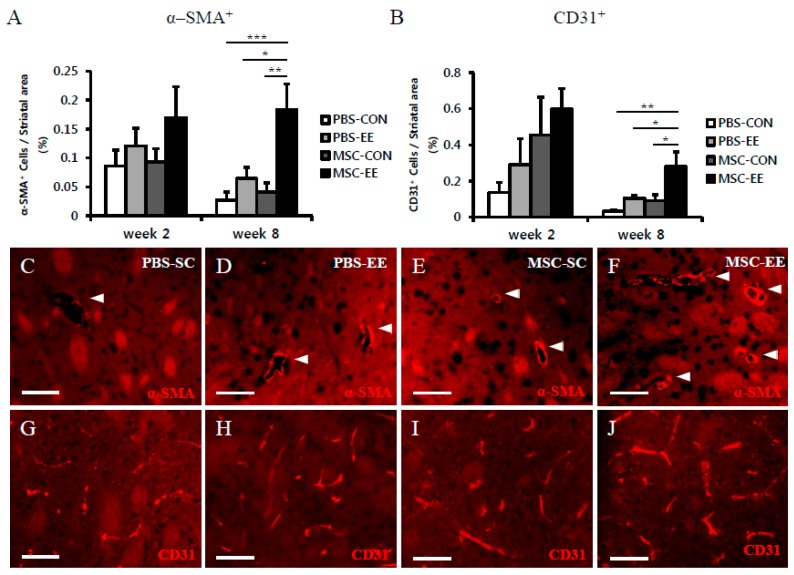

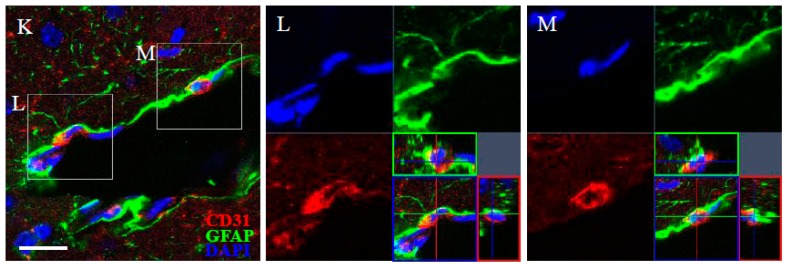
The Combination of MSCs and an EE enhanced endogenous angiogenesis in the striatum. (**A**–**J**) Two and eight weeks after an EE and MSC transplantation, the amounts of α-SMA^+^ cells (**A**) and CD31^+^ cells (**B**) were quantified using the MetaMorph Imaging System. Red color indicates α-SMA^+^ cells (**C**–**F**) and CD31^+^ cells (**G**–**J**). The density of: (**A**) striatal α-SMA^+^ cells (%) was significantly higher in the MSC-EE mice than in the PBS-CON, PBS-EE, and MSC-CON eight weeks after treatment (white arrows); and (**B**) CD31^+^ cells were significantly higher in striatum of the MSC-EE mice than in those of the other groups at eight weeks post-treatment (* *p* < 0.05, ** *p* < 0.01, and *** *p* < 0.001, respectively; *n* = 5 each); (**C**–**J**) Eight weeks after EE and MSC transplantation, representative images are shown. Scale bar = 50 μm; (**K**–**M**) CD31^+^ cells (red color) were surrounded with GFAP^+^ astrocytes (green color). Blue color indicates DAPI-positive nuclei. Scale bar = 20 μm. Values represent mean + SEM.

Based on our finding that the combination of an EE and MSCs resulted in increased astrocyte activation, we quantified the extent of angiogenesis induction in the neostriatum. We focused on this parameter because of the crucial role of astrocytes in maintaining the integrity of the BBB. The ability of MSC transplantation in combination with an EE to induce endogenous angiogenesis was assessed by histological analysis. Specifically, the densities of α-SMA-positive and CD31-positive cells were determined (Figure 3A–J). At two weeks post-treatment, the MSC-EE mice exhibited a modest tendency to exhibit enhanced angiogenesis, but no significant differences were observed among the other groups. Interestingly, the densities of α-SMA-positive and CD31-positive cells at two weeks post-treatment were higher than at eight weeks, but the differences were not statistically significant. The density of α-SMA^+^ cells in the MSC-EE group (0.18% ± 0.04%) was significantly higher than in the MSC-CON (0.04% ± 0.01%), PBS-EE (0.06% ± 0.02%), and PBS-CON groups (0.03% ± 0.01%) (Figure 3A,C–F; *p* < 0.01 compared with MSC-CON, *p* < 0.05 compared with PBS-EE, and *p* < 0.001 compared with PBS-CON). Likewise, the density of CD31^+^ cells was significantly increased in the MSC-EE (0.28% ± 0.08%) group compared with the MSC-CON (0.09% ± 0.03%), PBS-EE (0.1% ± 0.02%), and PBS-CON groups (0.03% ± 0.01%) (Figure 3B,G–J; *p* < 0.05 compared with MSC-CON and PBS-EE, and *p* < 0.01 compared with PBS-CON, respectively). Therefore, at eight weeks post-treatment, mice treated with an EE after MSC transplantation showed significant induction of angiogenesis in the neostriatum. We co-stained brain sections with GFAP and CD31, and the result showed that CD31^+^ cells were surrounded with GFAP^+^ astrocytes. This finding suggests that astrocytes were associated with endothelial cells (Figure 3K–M).

### 2.3. The Synergistic Effects of MSCs and an EE Upregulate Angiogenic Factors

**Figure 4:**
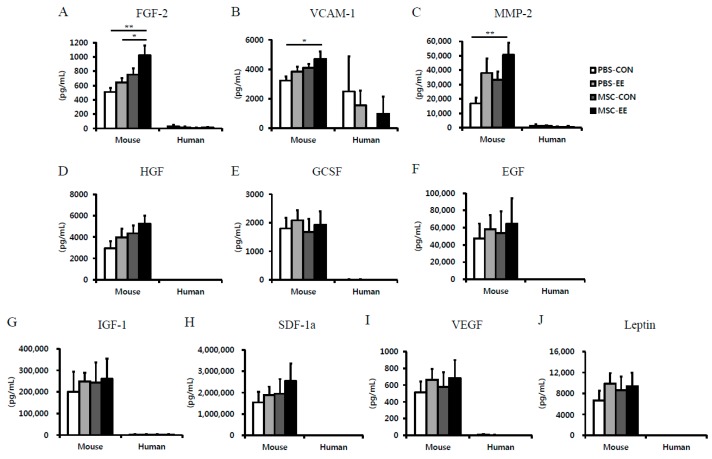
The angiogenic factors increased in the striatum of MSC-EE mice. (**A**–**J**) Two weeks after MSC transplantation and exposure to EE, the striatum were lysed and the levels of angiogenic proteins including FGF-2, VCAM-1, MMP-2, HGF, GCSF, EGF, IGF-1, SCF-1a, and Leptin were determined by a mouse-specific ELISA array; (**A**–**C**) The levels of FGF-2, VCAM-1 and MMP-2 were significantly higher in the right neostriatum of MSC-EE mice than it was in the PBS-CON tissues two weeks after treatment (* *p* < 0.05 and ** *p* < 0.01, *n* = 5, 4, 4, and 4 for PBS-CON, PBS-EE, MSC-CON, and MSC-EE, respectively). In a human-specific ELISA array, most of proteins were not detected. Values represent mean + SEM.

To identify the growth factors associated with MSC grafting and EE-induced astrocyte activation, the protein levels of mouse specific 10 candidate factors were measured in basal ganglia tissue samples using an array-based multiplex ELISA assay. Among tested the factors, FGF-2, vascular cell adhesion protein-1 (VCAM-1) and matrix metalloproteinase-2 (MMP-2) were significantly increased in the MSC-EE group compared with the PBS-CON groups at two weeks post-treatment (Figure 4A–C; *p* < 0.01 and *p* < 0.05). However, the following factors were not synergistically elevated in mice treated with the MSC-EE combination compared with mice in the other groups: epidermal growth factor (EGF), granulocyte colony-stimulating factor (GCSF), hepatocyte growth factor (HGF), insulin-like growth factor-1 (IGF-1), leptin, stromal cell-derived factor-1 (SDF-1), and VEGF. These results suggest that angiogenic factors such as FGF-2, VCAM-1, and MMP-2 secretion were enhanced through an EE after MSC transplantation, and that this role may involve angiogenesis and activated astrocytes. To identify the growth factors derived from which origin, mouse or human cells, we assessed these factors using a human-specific array-based multiplex ELISA assay. As a result, 10 candidate factors were not synergistically elevated. Furthermore, the levels of growth factors derived from mouse cells were expressed markedly higher than those from human-derived MSCs (Figure 4A–J). This result suggested that these factors were derived from mouse cells not human origin.

### 2.4. Astrocytes Co-Cultured with MSCs Secrete Angiogenic Factors

**Figure 5:**
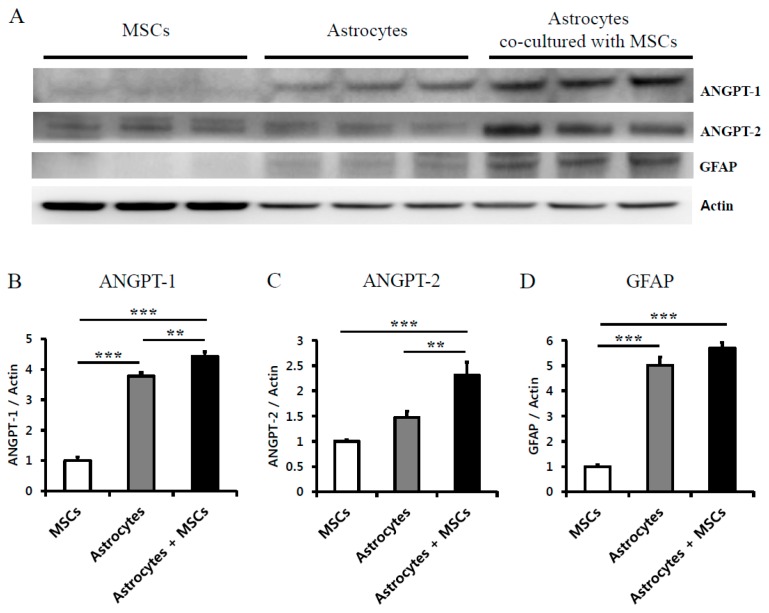
Stimulated astrocytes by MSCs released angiogenic factors: (**A**) representative images of Western blot analysis; (**B**,**C**) when astrocytes co-cultured with MSCs were cultured, angiogenic factors, such as ANGPT1 and ANGPT2, were significantly increased compared to cultured astrocytes alone and cultured MSCs alone (** *p* < 0.01 and *** *p* < 0.001, respectively, *n* = 3 each); and (**D**) the astrocytes co-cultured with MSCs and astrocytes cultured alone increased the expression of GFAP compared with MSCs groups (*** *p* < 0.001, *n* = 3 each). Values represent mean + SEM.

To confirm the effects of grafted MSCs on endogenous astroglial reaction in vivo, the angiogenic factors released by astrocytes co-cultured with MSCs were analyzed using Western blotting. The levels of ANGPT-1 (4.43 ± 0.15, *p* < 0.01) and ANGPT-2 (2.31 ± 0.25, *p* < 0.001) were significantly increased in astrocytes co-cultured with MSCs compared with astrocytes cultured alone (3.78 ± 0.12 and 1.48 ± 0.12, respectively) and MSCs (1.00 ± 0.12 and 1.00 ± 0.02, respectively) (Figure 5A–C). This result suggests that astrocytes would be stimulated by grafted MSCs and it released angiogenic factors including ANGPT-1 and ANGPT-2. We have quantified the levels of GFAP in activated astrocytes by MSCs, cultured astrocyte or MSCs alone to confirm stimulation by MSCs using Western blotting. As a result, the astrocytes co-cultured with MSCs and astrocytes cultured alone increased the expression of GFAP (5.70 ± 0.20 and 5.02 ± 0.33, respectively, *p* < 0.001) compared to MSCs cultured alone (1.00 ± 0.08, respectively).

### 2.5. Rotarod Performance and Grip Strength Results Indicate that an EE Synergistically Improves Neurobehavioral Function after MSC Transplantation

**Figure 6:**
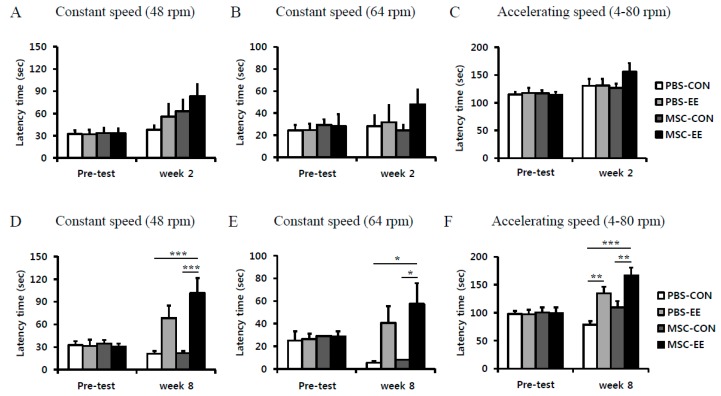
The combination of MSCs and EE synergistically improved motor function. (**A**–**F**) The rotarod tests were performed at two and eight weeks post-treatment; (**A**–**C**) Two weeks after treatment, MSC-EE mice expressed a tendency to exhibit enhanced motor function, but no significant differences were observed among the groups; (**D**–**F**) Eight weeks after the treatment, the rotarod tests at constant (48 and 64 rpm) and accelerating (4–80 rpm) speeds showed that the MSC-EE mice were significantly improved compared to those of MSC-CON and PBS-CON (* *p* < 0.05, ** *p* < 0.01, and *** *p* < 0.001, *n* = 15 each). Values represent mean + SEM.

To determine whether MSCs and an EE can restore neurobehavioral function, we performed rotarod tests both at constant (48 and 64 rpm) and accelerating (4 to 80 rpm) speeds at two and eight weeks post-treatment. The MSC-EE mice exhibited a tendency of improvement in rotarod performance at two weeks post-treatment when the test was performed at a constant speed of 48 or 64 rpm and also at an accelerating speed; but no significant differences were observed among the groups (Figure 6A–C). Importantly, at eight weeks post-treatment, the MSC-EE mice (101.5 ± 19.8 s) exhibited a significantly longer latency period than the mice in the other groups: MSC-CON (21.9 ± 3.0 s), PBS-EE (68.5 ± 16.2 s) and PBS-CON (21.0 ± 3.5 s) at 48 rpm (Figure 6D; *p* < 0.001 compared with MSC-CON and PBS-CON groups). At 64 rpm speed at eight weeks post-treatment, the MSC-EE mice (57.4 ± 18.4 s) exhibited a significantly longer latency period than the mice in the other groups: MSC-CON (8.0 ± 2.6 s), PBS-EE (40.5 ± 14.9 s) and PBS-CON (5.4 ± 1.6 s) (Figure 6E; *p* < 0.05 compared with MSC-CON and PBS-CON groups). Similarly, the MSC-EE mice had the best rotarod test results (166.7 ± 14.2 s) among the groups at eight weeks post-treatment when the test was performed at an accelerating speed, compare to MSC-CON (109.6 ± 11.3 s) and PBS-CON mice (78.6 ± 6.4 s) (Figure 6F; *p* < 0.01 and *p* < 0.001 compared with MSC-CON and PBS-CON groups, respectively). PBS-EE (134.6 ± 12.2 s) group also significantly increased latency time compared with PBS-CON (Figure 6F; *p* < 0.01 compared with PBS-CON group). These results suggest that MSC transplantation alone is not sufficient for functional recovery, and that the combination of an EE and MSCs could enhance sustained neurobehavioral improvement.

**Figure 7:**
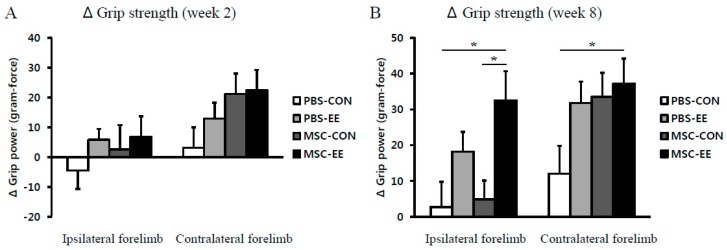
The combination of MSCs and EE synergistically improved grip power. Grip strength test was performed in ipsilateral (**right**) and contralateral (**left**) forelimbs two and eight weeks after treatment. (**A**) Two weeks after treatment, the MSC-EE mice expressed a tendency to exhibit enhanced motor function, but no significant differences were observed among the groups; (**B**) The MSC-EE mice were increased the grip strength in both limbs (* *p* < 0.05, *n* = 15 each). Values represent mean + SEM.

We next evaluated forelimb grip strength at two and eight weeks post-treatment. No significant differences were observed among the groups at two weeks post-treatment (Figure 7A). However, at eight weeks post-treatment, the grip power in the contralateral hemiplegic limb relative to its preoperative value was significantly improved in MSC-EE mice (37.2 ± 7.1 gram-force) compared with the PBS-CON controls (12.0 ± 7.7) (Figure 7B; *p* < 0.05 by one-way ANOVA). Interestingly, the grip power of the ipsilateral control limb was enhanced only in the MSC-EE mice (32.5 ± 8.2), not in the MSC-CON (4.8 ± 5.2), PBS-EE (18.2 ± 5.5), or PBS-CON mice (2.8 ± 7.0) (Figure 7B; *p* < 0.05 compared with MSC-CON and PBS-EE groups). This result suggests that the combination of EE and MSC transplantation was effective in improving muscle power after unilateral ischemic brain damage.

## 3. Discussion

The present study demonstrated that an EE exerts synergistic effects on hypoxic-ischemic brain injury after MSC transplantation. Activated astrocytes released angiogenic factors including FGF-2, VCAM-1, and MMP-2 without forming glial scars. Furthermore, we found that astrocytes exhibited enhanced angiogenesis to induce functional recovery. Since our data indicate that an EE enhances angiogenesis and astrocyte-mediated secretion of angiogenic factors in the presence of MSC stimulation, we propose that an EE and MSC transplantation are effective treatments for cerebral palsy (CP).

Hypoxic-ischemic brain injury is a major cause of damage in the fetal and neonatal periods and occurs in 2~3 of every 1000 full term infants [27,28]. This type of brain injury can result in permanent disabilities such as CP, mental retardation, learning disabilities, and epilepsy [28,29]. Moreover, the treatments for the chronic stages of these diseases are extremely limited, because of the difficulty in inducing regeneration.

Stem cell therapy (e.g., neural stem/precursor cells and MSCs) is another potential treatment that has been used to promote regeneration in brain damage. Of all stem cell types, MSCs are abundantly harvested from bone marrow, umbilical cord blood, or adipose tissue and can be used in tissue engineering [6]. Recent evidence suggests that MSCs can produce several beneficial growth factors (e.g., HGF, IGF-1, VEGFA, BDNF, and NGF) [30,31,32], thereby affecting paracrine mechanisms rather than cell replacement and direct tissue regeneration. Systemic injection of MSCs has been shown to promote functional recovery in spinal cord injury [33]; however, direct injection of these cells did not significantly enhance behavioral functions [3]. Since grafted cells are often limited to homing by the immune system, cell therapy alone may not be sufficient for functional restoration. Thus, cell transplantation alone may be not sufficient to achieve stable beneficial effects for neurorestoration because of engraftment limitations [34].

The beneficial effects of physical training as rehabilitation, such as an EE, have been reported in animal studies. An EE is known to induce the release of neurotrophic factors (e.g., BDNF, NGF, GDNF, and FGF-2) that have important roles in restoration [4,35,36,37], increasing neuronal plasticity [5,38] and reducing lesion size [39]. Several studies have revealed that an EE can induce angiogenesis via angiogenic factors and can also stimulate astrocyte activation, which supports angiogenesis [4,40]. Similarly, we have demonstrated the beneficial effects of an EE with respect to neurogenesis, angiogenesis, synaptogenesis, and functional improvement in normal and injured mice [3,4,5].

One previous study reported that an EE enhances the integration and neurogenic fate of transplanted cells [3]. Therefore, the EE has been expected to become part of therapies that can overcome the limitations of cell transplantation. In addition with neurogenesis, recent studies have shown that astrocytes, which are the most abundant cells in the brain, play important roles in the transport of glutamate, maintenance of homeostasis and the BBB, modulation of inflammation, and secretion of neurotrophic factors [7,41,42]. When the brain is damaged, astrocytes are transformed to their reactive state. If this state persists, glial scars are formed, followed by astrocyte retraction of their endfeet connections to blood vessels. However, since angiogenesis is an essential part of brain injury [4], astrocytes are believed to be important for recovery. Recent studies have suggested that MSC transplantation can stimulate astrocytes, which release beneficial factors such as GDNF, VEGF, FGF-2, and BDNF [21,22,23,24,25,26]. Moreover, MSCs have been shown to inhibit the formation of glial scars in a rat model of stroke [22,32,43].

In the present study, among the protein levels of ten candidate factors associated angiogenesis, FGF-2, VCAM-1, and MMP-2 were significantly increased in the basal ganglia of MSC-EE mice. However, most of proteins were not detected in human Quantibody^®^ array. This result suggested that these factors were derived from mouse cells not human origin.

In our mouse model of hypoxic-ischemic brain injury, we found that activated astrocytes after MSC treatment were associated with angiogenesis and functional recovery. In addition, astrocytes cultured in vitro with MSCs released more angiogenic factors, including ANGPT1 and ANGPT2, than astrocytes cultured alone. Furthermore, Gao et al. suggested that MSCs can increase astrocyte survival via the phosphoinositide 3-kinase/threonine protein kinase pathway and enhance the expression of a gap junction protein, connexin 43, which is derived from astrocytes in vitro [22,44,45]. Therefore, astrocyte-mediated growth factors stimulated by MSCs might help restore BBB integrity.

Interestingly, astrocyte activation and secretion of angiogenic factors were robustly increased at earlier stage (two weeks after treatment) and angiogenesis was exhibited at later stage (eight weeks after treatment). This result suggests that beneficial environment with upregulation of angiogenic factors has driven to be restored by an EE and MSC transplantation, and thereafter angiogenesis was significantly increased, consequently resulting in functional recovery in the neurobehavioral outcomes. Our results show that motor function and forelimb muscle power were enhanced by an EE after MSC treatment, suggesting that neurological behavior was synergistically improved with the combination treatment of cell therapy and an EE. We also found that endogenous angiogenesis in parallel with upregulation of angiogenic factors including FGF-2, VCAM-1, MMP-2, ANGPT-1 and ANGPT-2 released from activated astrocytes in vivo and in vitro, enhances functional recovery through synergistic effects of the EE and MSC combination in mice with hypoxic-ischemic brain injury.

## 4. Materials and Methods

### 4.1. Animals

Mice were housed under climate-controlled conditions with a 12-h light/dark cycle and were provided standard food and water ad libitum. Mice of both sexes were used. All procedures were reviewed and approved by the Animal Care and Use Committee of Yonsei University College of Medicine (identification code: 2013-0209) on 11 July 2013.

### 4.2. Neonatal Hypoxic-Ischemic Brain Injury

Permanent ischemic brain damage was induced in 7-day-old CD-1^®^ (ICR) mice by unilateral right carotid artery ligation. Hypoxic brain injury (8% O_2_ for 90 min) was induced as previously described [3]. Body temperature was maintained at 37 °C within the hypoxic chamber. One week after HI brain injury, a scalp incision was made to identify the brain lesion in the posterolateral area of the right hemisphere. The presence and extent of brain injury in all subjects were assessed through the semi-transparent skull. Animals with severe brain lesions covering more than 50% of the unilateral hemisphere were excluded to eliminate potential sampling error due to volumetric changes in the neostriata.

### 4.3. Cell Transplantation

At Postnatal Week 6 (P42), mice were anesthetized with ketamine (100 mg/kg) and xylazine (10 mg/kg) by intraperitoneal (i.p.) injection. Mice were randomly assigned to one of two groups for treatment with either MSCs or PBS. The mice received an intrastriatal injection of MSCs (1 × 10^5^ cells, 2 μL volume, 0.01 μL/s infusion rate) or PBS using stereotaxic coordinates (AP +0.5 mm from bregma; ML −1.5 mm from bregma; DV −3.5 mm from the dura). Body temperatures were maintained at 37 °C in a heating chamber during the recovery period.

### 4.4. Grouping and Experimental Housing

A total of 116 mice were recruited in this study. After stereotaxic surgery, 56 mice were randomly assigned in four groups for 2 weeks cohort: PBS-CON (*n* = 13), PBS-EE (*n* = 13), MSC-CON (*n* = 15), and MSC-EE (*n* = 15). For 8 weeks cohort, 60 mice were randomly assigned in four groups: PBS-CON (*n* = 15), PBS-EE (*n* = 15), MSC-CON (*n* = 15), and MSC-EE (*n* = 15). Whereas the SC controls were housed for the same duration in a standard cage (27 × 22.5 × 14 cm^3^) without social interaction (3–4 mice/cage), the EE mice were housed in a spacious cage (86 × 76 × 31 cm^3^) containing novel objects such as tunnels, shelters, toys, and running wheels for voluntary exercise. In addition, the EE mice were allowed social interaction (12–15 mice/cage) for up to 2 months (Figure 1D). All animals were housed in a facility accredited by the Association for Assessment and Accreditation of Laboratory Animal Care (AAALAC) and were given food and water ad libitum with alternate 12-h light/dark cycles, according to animal protection regulations. The experimental procedure was approved by the relevant institutional review board (IRB). A schematic timeline of the experiment from birth to 14 weeks of age is provided in Figure 1A.

### 4.5. Behavioral Assessment

ROTAROD PERFORMANCE: A rotarod test was used to assess motor coordination and balance. All animals received a preoperative performance evaluation at 5–6 weeks of age. The rotarod tests were then performed at 2-week intervals until 8 weeks post-transplantation using constant speed (48 and 64 rpm) and accelerating speed (4–80 rpm) paradigms. The latency time of each mouse to fall from the rod was measured twice during each test; individual tests were terminated at a maximum latency time of 300 s [3].

GRIP STRENGTH TEST: A grip strength test was performed using the SDI Grip Strength System (San Diego Instruments Inc., San Diego, CA, USA), which includes a push-pull strain gauge. A triangular piece of metal wire 2 mm in diameter was used as the grip bar. Each animal was held near the base of its tail and was moved in close proximity to the bar until the animal could grip the bar with its forepaws. Peak force was automatically registered in gram-force by the apparatus. The mean peak force of three trials was used for analysis [3].

### 4.6. Cell Culture: Co-Culture System

Mouse astrocytes (Astrocyte Type 1 clone, ATCC, CRL-2541) and MSCs were cultured in Dulbecco’s modified Eagle’s medium-H (DMEM-high glucose; No. 30-2002, American Type Culture Collection (ATCC), Manassas, VA, USA) and DMEM-L (DMEM-low glucose; No. 11885-084, Gibco, Carlsbad, CA, USA) supplemented with 10% fetal bovine serum and 1% antibiotics (P/S). MSCs (1 × 10^5^ cells) were cultured on the upper side of a Falcon 0.4 μm cell-culture insert (No. 353090, BD Bioscience, San Jose, CA, USA) in 2 mL medium, with 5 × 10^5^ astrocytes cultured on the opposite side of the insert plate in 3 mL medium, in a total of 5 mL medium (*n* = 3/group). As negative controls, MSCs and astrocytes were cultured alone. After the cells had adhered to the insert well, they were washed with Hank’s Balanced Salt Solution (HBSS, Gibco) four times, and then the media were replaced with serum-free media that can culture astrocytes. After 48 h, protein was extracted from the cells for Western blot analysis.

### 4.7. Immunohistochemistry

Immunohistochemistry was performed as previously described [46]. Briefly, animals were euthanized and perfused with 4% paraformaldehyde (PFA). The harvested brain tissue samples were cryosectioned with a slice thickness (16-μm), and immunohistochemistry staining was performed on four sections over a range of 128 μm. Sections were stained with primary antibodies against GFAP (1:400, Abcam, Cambridge, UK), CS-56 (1:200, Abcam), a-SMA (1:200, Abcam), and CD31 (1:200, BD Bioscience, San Jose, CA, USA). For visualization, the following secondary antibodies were used: Alexa Fluor^®^ 594 goat anti-rat (1:400, Invitrogen, Carlsbad, CA, USA), Alexa Fluor^®^ 594 goat anti-rabbit (1:400, Invitrogen), and Alexa Fluor^®^ 488 goat anti-mouse (1:400, Invitrogen). Tissue samples were mounted on glass slides with fluorescent mounting medium containing 4’,6-diamidino-2-phenylindole (DAPI; Vectorshield, Vector, Burlingame, CA, USA). The stained sections were then analyzed using a confocal microscope (LSM700, Zeiss, Gottingen, Germany) and the MetaMorph Imaging System (Molecular Device, Sunnyvale, CA, USA). Blood vessel density was evaluated using a fluorescence microscope (BS51, Olympus, Tokyo, Japan) and the MetaMorph Imaging System. Images of glial scarring were captured using a fluorescence microscope (Axio Imager M2, Zeiss); scarring density was evaluated using ZEN Imaging Software (Blue edition, Zeiss).

### 4.8. Assessment of Growth Factors in the Striatum

To identify growth factors that are regulated by MSC transplantation and/or an EE, neostriata were separated from the surrounding brain tissue and lysed in 200 μL of cold RIPA buffer (50 mM Tris-HCl, pH 7.5, 1% Triton X-100, 150 mM NaCl, 0.1% sodium dodecyl sulfate (SDS), 1% sodium deoxycholate) containing a protease inhibitor cocktail (Sigma-Aldrich, St. Louis, MO, USA). Tissue lysates were then centrifuged at 13,000× *g* for 15 min at 4 °C. The supernatants were harvested, and the protein concentrations were determined using a protein assay kit (Bio-Rad, Hercules, CA, USA). An array-based multiplex ELISA assay (Mouse Quantibody^®^ array, RayBiotech, Norcross, GA, USA) was used to determine which of the following 10 mouse cytokines or growth factors were detectable in the neostriata: FGF-2, EGF, GCSF, HGF, IGF-1, LEP, MMP-2, SDF-1a, VCAM-1, and VEGF. Expression of angiogenic factors was detected using an array scanner (Gene PIX™ 4000B, Axon instruments, Sunnyvale, CA, USA).

### 4.9. Western Blotting

Harvested cells were lysed in 50 μL cold lysis buffer (50 mM Tris-HCl, pH 7.5, 1% Triton X-100, 150 mM NaCl, 0.1% sodium dodecyl sulfate (SDS), 1% sodium deoxycholate) with a protease inhibitor cocktail (Sigma-Aldrich, St. Louis, MO, USA). Lysates were then centrifuged at 13,000× *g* for 15 min at 4 °C. The supernatants were harvested, and the protein concentrations were determined using a protein assay kit (Bio-Rad Laboratories). Proteins (20 μg) were solubilized in sample buffer (60 mM Tris-HCl, pH 6.8, 14.4 mM β-mercaptoethanol, 25% glycerol, 2% SDS, and 0.1% bromophenol blue), denatured for 10 min at 90 °C, and subjected to electrophoresis on a 10% SDS polyacrylamide gel. The resolved proteins were then transferred onto a 0.2 mm InvitrolonTM polyvinylidene difluoride (PVDF; Invitrogen) membrane using an XCell IITM Blot Module (Invitrogen). The membranes were blocked for one hour in TBST (Tris-buffered saline (10 mM Tris-HCl, pH 7.5, 150 mM NaCl) containing 0.05% Tween 20) supplemented with 5% nonfat dry milk (Bio-Rad Laboratories) at room temperature and washed three times with TBST. After blocking, the membranes were incubated at 4 °C overnight in TBST supplemented with 5% nonfat dry milk and the appropriate primary antibodies. Membranes were incubated with antibodies against ANGPT1 (1:500, Abcam), ANGPT2 (1:500, Abcam), GFAP (1:500, Abcam), and Actin (1:1000, Santa Cruz Biotechnology Inc., Dallas, TX, USA). The blots were then washed three times with TBST and incubated for one hour with horseradish peroxidase-conjugated secondary antibodies (1:3000, Santa Cruz Biotechnology) at room temperature. After washing three times with TBST, immunoreactive bands were visualized with an enhanced chemiluminescence detection system (Amersham Pharmacia Biotech, Little Chalfont, UK).

### 4.10. Statistical Analysis

All data are expressed as mean ± SEM. Differences between groups were analyzed using one-way analysis of variance (ANOVA) followed by a post-hoc Bonferroni comparison. All analyses were performed using SPSS software (IBM Corporation, Armonk, NY, USA; version 20.0). A *p* value < 0.05 was considered statistically significant.

## 5. Conclusions

Our findings demonstrate that MSC transplantation and an EE synergistically promoted neurological function by enhancing endogenous angiogenesis and astrocyte activation and upregulating angiogenic factors including FGF-2, VCAM-1, and MMP-2. Similarly, we found that MSC-activated astrocytes secreted angiogenic factors in vitro. Therefore, our results indicate that a rehabilitative strategy combined with a cell-based therapy may be an effective combination for treating angiogenesis in CP. Moreover, an EE could be a useful strategy for enhancing other desirable effects from cell therapy in additional neurological diseases.
